# Psychosocial Work Environment, Occupational Stress, and Health Risk Profiling Among Rotational Workers at a Mining and Processing Enterprise in Kazakhstan: An Integrated Assessment

**DOI:** 10.3390/healthcare14131888

**Published:** 2026-06-29

**Authors:** Yertay Otarov, Zhenisbek Zharylkassyn, Alexey Alexeyev, Chingiz Ismailov, Zhanbol Sabirov, Magzhan Tilemissov, Almagul Shadetova, Didar Okassov, Ulbala Shaikhattarova, Nazgul Izdenova

**Affiliations:** 1 National Center of Labour Hygiene and Occupational Diseases, Karaganda 100017, Kazakhstan; otarov_kgmu@mail.ru (Y.O.); alexeyev.ncgtpz@mail.ru (A.A.); zap.audacious@gmail.com (Z.S.); tilemisov.m@mail.ru (M.T.); shadetova@kgmu.kz (A.S.); okasovdb@mail.ru (D.O.); 2 School of Public Health, Karaganda Medical University, Karaganda 100012, Kazakhstan; zhenisbekz@mail.ru (Z.Z.); nazgul.abenova@mail.ru (N.I.); 3 Department of Public Health and Scientific Research, Khoja Akhmet Yassawi International Kazakh-Turkish University, Turkestan 161200, Kazakhstan; ulbala.shaikhattarova@ayu.edu.kz

**Keywords:** occupational health risk, psychosocial work environment, occupational stress, temporary disability, rotational workers, mining industry, Kazakhstan

## Abstract

**Highlights:**

**What are the main findings?**

- Psychosocial profiles were less favorable for production units than for administrative staff, with the enterprise recording 4971 eligible sickness absence episodes and 61,472 lost workdays over 2020–2024.
- The integrated working conditions index was positively associated with sickness absence and occupational stress, whereas the health index was inversely associated with psychosocial well-being, supporting an integrated occupational risk pattern.

**What are the implications of the main findings?**

- Occupational risk assessment in industrial enterprises may be incomplete if psychosocial factors are assessed separately from hygiene- and health-related indicators.
- Integrating psychosocial monitoring, stress screening, and sickness absence metrics into routine occupational health surveillance may improve prevention prioritization and support healthier workplace management.

**Abstract:**

**Background:** Occupational health risk assessment in industrial enterprises has traditionally focused on physical, chemical, and ergonomic hazards, while psychosocial working conditions have often been assessed separately from routine occupational surveillance. The aim of this study was to examine whether integrating working conditions, the psychosocial work environment, occupational stress, and temporary disability indicators provide a more informative health risk profile in an industrial setting. **Methods:** An analytical observational study was conducted at a mining and processing enterprise in Kazakhstan. This study used three data sources: 5429 temporary disability records for 2020–2024, workplace assessments covering 188 job positions, and psychosocial survey data from 392 employees. Occupational stress was evaluated in annual PSS-25 screening waves conducted in 2023 (n = 133), 2024 (n = 133), and 2025 (n = 134). The author-developed psychosocial questionnaire showed acceptable internal consistency (Cronbach’s α = 0.82). **Results:** During the five-year period, 4971 eligible temporary disability episodes and 61,472 lost workdays were recorded. Psychosocial profiles were less favorable in production units than in administration, and mean PSS-25 values remained relatively stable across the years. The Integral Index of Working Conditions (Iwc) was positively associated with temporary disability indicators and occupational stress, whereas the Integral Health Index (Ihr) was inversely associated with psychosocial well-being. **Conclusions:** The findings suggest that occupational risk assessment remains incomplete when psychosocial factors are excluded or treated separately. Integrating hygienic, psychosocial, stress-related, and medical-statistical indicators may improve the prioritization of preventive measures and support healthier workplace management in industrial enterprises.

## 1. Introduction

The world of work has undergone an unprecedented transformation in the past several decades, with structural changes occurring under the impetus of economic globalization, technological advances, and growing labor market integration. Occupational health as such has evolved to include psychosocial as well as social and workforce factors regarding the biological, chemical, human, and psychological determinants of workers’ well-being [1,2]. Whereas high-income countries are front runners in this shift, the emerging middle-income countries are carrying more of a double burden: traditional occupational hazards, the risks of precarious work, and unsafe work are enduring factors, now combined with increasing exposure to psychosocial factors—job insecurity, job precariousness, work intensification, and unstable job mobility—that were classically perceived as being characteristic of more affluent economies. Hence, psychosocial occupational health is becoming more essential for occupational risk assessment in industrial sectors with high rates of technological and organizational change in middle-income countries [2].

The traditional occupational safety systems in industrial enterprises have emphasized measurable physical, chemical, and ergonomic hazards. Nevertheless, psychosocial risk factors such as perceived organizational injustice, a deficiency of workplace support, chronic occupational stress, and a detrimental organizational climate are now understood as considerable determinants of health, temporary disability, and long-term functional impairment among workers [1,2,3,4,5]. Current guidance documents emphasize that psychosocial risk management should be fully incorporated into occupational safety and health systems, rather than a secondary or supplemental concern [3,4].

The last decade has seen psychosocial risk surge to the forefront of occupational health, and with good reason. We now know that workers’ health is not equal to their exposure levels to dust, noise, or chemical agents. How work is organized matters no less. Whether employees really know what they are doing, whether they feel supported by management, and whether they exercise any meaningful control over their work—all of these variables shape the psychosocial working environment, and thus, help to underpin the well-being of staff throughout the day [6].

Stresses in this light are not a matter of single-minded fortitude but are rather reflective of the conditions that people experience day after day at work. The evidence from theoretical studies and empirical studies conducted in occupational medicine is consistent in its conclusion that heavy job stressors, weak decision-making freedom, effort–reward conflict, and reduced social support contribute to poor physical and mental well-being [6,7]. Recent research further evidence that psychosocial stress not only adds to an individual’s subjective distress but also heightens temporary disability rates and the overall health burden [7,8,9,10,11,12]. Accordingly, risk assessment models based on old-fashioned occupational hazards alone may massively underestimate the extent of health risks in the workplace.

The importance of a comprehensive assessment method is especially highlighted in the mining and processing industries, where there is concurrent exposure of workers to noise, dust, vibration, physical exertion, and various organizational and psychological stressors. In this workgroup, bronchopulmonary diseases are consistently linked to serious health issues, highlighting a dual toll of physical exposures on worker well-being [13,14,15]. More specifically, research performed in Kazakhstani mining industries has established that extended exposure periods to industrial dust can be linked to alterations in cellular immunity, i.e., inhibition of CD3+ and CD4+ T-cells, increased neutrophil phagocytic activity, and stress-related systemic health consequences of production-induced exposures [16]. The combined risk profiles that are most important for directed preemptive intervention cannot be determined when these domains are analyzed in isolation.

This is especially the case in industrial environments and blue-collar work contexts, where physical symptoms of hazards and management stress tend to be connected. Workers on the assembly line are accustomed to noise, vibration, wear and tear, and high workloads; have little input into what happens to them; and experience gaps in the lines of communication between management and the shop floor. Studies from these settings have consistently demonstrated that a negative psychosocial environment, which is worse than a psychological or even individual problem, does great damage (undermines job satisfaction, reduces organizational commitment, and elevates workers’ anxiety) [10,11,17]. Thus, psychosocial circumstances are not marginal in the industrial work environment but add to the burden of stressors from classical accidents or workplace hazards.

The stakes are even more obvious when we investigate the long-term sustainability of the workforce. Previous studies covering older workers over time showed that a combination of high physical demand and poor psychosocial conditions makes premature exit from work more likely—often well before the statutory retirement age has been reached. For enterprises with experienced workers who still perform highly physically demanding work and experience higher organizational pressure, this is not only an issue of policy but practically a pressing business concern. The above evidence collectively forms a strong argument for an occupational health model that combines physical working conditions, psychosocial circumstances, occupational stress, and health effects and is multi-leveled (single analysis), to be assessed as a holistic health model [18].

The current study was developed to fill this methodological gap. A unified analytical framework was built from workplace condition assessment, psychosocial work environment characterization, occupational stress screening, and temporary disability analysis. The main objective was to evaluate whether integrating working conditions, psychosocial indicators, occupational stress, and health outcome data optimizes the occupational risk profiling of an industrial enterprise. The specific objectives were to (1) characterize the burden of temporary disability; (2) analyze working conditions and the psychosocial work environment; (3) assess the degree of occupational stress; and (4) explore the interrelationships between these domains.

## 2. Materials and Methods

### 2.1. Study Design and Setting

This analytical observational study was conducted at a mining and processing enterprise in Kazakhstan and comprised four empirical blocks: temporary disability analysis, occupational risk assessment at the workplace, psychosocial work environment assessment, and integrated statistical analysis of the resulting indicators.

### 2.2. Participants and Sampling

The occupational risk assessment involved 188 workplaces of the enterprise’s main divisions. The psychosocial survey included 392 employees from the enterprise’s main occupational groups, including technological units, open-pit operations, services, and administration. Inclusion criteria: Age 18–60 years (working age in Kazakhstan), a rotational shift work schedule (11 h day shift, 11 h night shift, 15-day rotation cycle, repeating), direct occupational contact with industrial hazards (technological production units, open-pit mining units, service/maintenance divisions) or no such contact (administrative and engineering–technical personnel), and completion of the mandatory annual occupational health medical examination. Exclusion criteria: A registered diagnosis of occupational or chronic disease prior to study enrollment, health conditions requiring comprehensive investigation or medical registry observation, loss of work capacity in the year prior to enrollment, occupational position change within 2 years of the study period, work interruptions (labor leave, professional development courses, other absences) exceeding 6 months in the 6-month period preceding survey participation, and refusal to provide written informed consent.

Of 852 eligible male workers employed in occupations with potential industrial exposure (n = 534 in production/mining; n = 318 in auxiliary/administrative), 392 employees completed the psychosocial survey component (response rate: 73.4%). The final analytical sample was distributed across four occupational groups: technological production units (n = 96), open-pit mining units (n = 193), service and maintenance divisions (n = 55), and administration/engineering–technical personnel (n = 48). The mean age (40.8 ± 8.4 years) and mean occupational tenure (12.1 ± 9.2 years) of the study sample correspond to the demographic profile of workers in Kazakhstan’s mining and mineral processing industry, supporting the representativeness of the findings (Table 1).

### 2.3. Temporary Disability Data

The temporary disability block consisted of 5429 medically certified temporary disability records for 2020–2024. After application of the analytical processing rules used in the study dataset, 4971 eligible episodes were retained for the final calculations. Temporary disability was characterized using four standard occupational health indicators: P1, the number of cases per 100 employees; P2, the number of disability days per 100 employees; P3, the mean duration of one episode; and P4, the number of affected workers per 100 employees.

### 2.4. Integral Indices

The Integral Index of Working Conditions (Iwc) was derived from a hygiene assessment of workplace factors. To construct the index, hygiene classes were converted into numerical scores (Class 2 = 0; Class 3.1 = 1; Class 3.2 = 2; Class 3.3 = 3; Class 3.4 = 4), and a weighted average score was then calculated across the relevant workplace factors. Higher Iwc values indicated less favorable working conditions. The Integral Health Index (Ihr) was derived from normalized temporary disability indicators using the weighted combination 0.3 × P1 + 0.3 × P2 + 0.2 × P3 + 0.2 × P4, with higher values corresponding to a less favorable health profile.

The construction of the composite indices (Iwc and Ihr) followed a formalized additive model. The weighting scheme for the Integral Index of Working Conditions (Iwc) was derived from the hazard classes (Classes 1–4) defined by Guideline R 2.2.1766-03 “Guidelines on occupational risk assessment for workers’ health. Organizational and methodological aspects, principles, and criteria”. This ensures that the weights reflect established hygienic principles of cumulative occupational burden rather than arbitrary assignments. For the psychosocial environment components, a unit-weighting approach was adopted, where each domain contributed equally to the final score, ensuring methodological transparency and alignment with exploratory research standards.

### 2.5. Psychosocial Assessment of the Work Environment

The psychosocial work environment was assessed using an author-developed questionnaire tailored to the industrial occupational context. The analytical version comprised 21 items grouped into three conceptual domains: social responsibility and support, social justice and work organization, and employee well-being/social package. Responses were transformed into domain-specific and integral indices on a 0–100 scale, with higher values indicating more favorable psychosocial conditions.

The structural validity of the 21-item questionnaire was examined using exploratory factor analysis (EFA) with maximum likelihood extraction and varimax rotation. The Kaiser–Meyer–Olkin (KMO) measure of sampling adequacy was 0.873, and Bartlett’s test of sphericity indicated significance (χ^2^(210) = 3247.6, *p* < 0.001). A three-factor solution was retained based on eigenvalues >1.0 and the scree plot, explaining 68.4% of the total variance. Factor loadings after varimax rotation ranged from 0.52 to 0.80 for the primary items, with no significant cross-loadings (>0.40) across factors. The three factors corresponded to the theoretically predefined domains: social responsibility and support (9 items), organizational justice and working conditions (7 items), and social benefits and employee well-being (5 items). Exploratory factor analysis was used to explore the structural validity; confirmatory factor analysis on an independent sample has not yet been performed.

### 2.6. Assessment of Occupational Stress

Occupational stress was assessed using the PSS-25 scale (Perceived Stress Scale, Industrial version), a 25-item instrument with 5-point Likert response options (0 = “never,” 1 = “rarely,” 2 = “sometimes,” 3 = “often,” 4 = “constantly”). The PSS-25 was selected because it provides a more comprehensive assessment of work-related stress manifestations—including subjective perception of workload intensity, emotional tension, anxiety, fatigue, and occupational burnout symptoms—compared with shorter versions (PSS-10, PSS-14). This expanded item pool is particularly suited to mining and processing work environments where multiple occupational stressors operate simultaneously.

The PSS-25 survey was conducted during annual medical examinations in 2023–2025 among workers of the main occupational groups, with repeated assessment of the same cohort performed whenever feasible. The temporal structure of the datasets reflected the logic of enterprise health surveillance: sickness absence data were first analyzed for a completed calendar year, and psychosocial and stress assessments were then administered during the following year’s medical examinations. Thus, temporary disability data for 2022 were analytically linked with questionnaire data collected in 2023, data for 2023 were linked with stress assessments in 2024, and data for 2024 were linked with stress assessments in 2025. This design did not create a strictly synchronous dataset, but it allowed the study to examine whether the prior year’s morbidity burden corresponded to subsequent stress and psychosocial profiles observed during routine occupational health monitoring. In the present analysis, results were considered as continuous scores and as four ordered categories (low, moderate, high, and very high stress) according to the interpretation scheme used in the enterprise screening protocol. The annual stress subsamples comprised 133 employees in 2023, 133 employees in 2024, and 134 employees in 2025.

### 2.7. Statistical Analysis

Descriptive statistics were used to summarize quantitative and categorical variables. Between-group comparisons for psychosocial indicators were performed using Student’s *t*-test or one-way analysis of variance (ANOVA), as appropriate. Exploratory factor analysis (EFA) was used to examine the structure of the author-developed psychosocial questionnaire, with Cronbach’s alpha used to assess internal consistency. Convergent validity was examined through correlations between the integral psychosocial index and PSS-25 scores. To determine the independent effect of the psychosocial work environment on occupational stress, a multivariable linear regression analysis was performed on individual-level data. This model specifically adjusted for key confounding variables, including age, sex, and professional tenure. To evaluate the potential for common method bias, Harman’s single-factor test was performed using all self-reported variables. The single-factor solution accounted for 26.3% of the total variance, which is well below the 50% threshold, suggesting that the associations observed in the study are not driven by common method variance.

For health indicators (morbidity rates) available only as aggregated unit-level data, analysis was restricted to descriptive comparisons and trend assessment to avoid the risk of ecological fallacy. Bivariate associations were examined using Pearson or Spearman correlation coefficients depending on the distribution of data This exploratory study was designed as an exploratory integrated assessment; therefore, all reported associations should be interpreted as descriptive and non-causal, given the cross-sectional and cross-level nature of the data.

### 2.8. Ethical Considerations

Participation in the survey was anonymous and voluntary. Written informed consent was obtained from all employees involved in the survey component of the study.

## 3. Results

### 3.1. The Profile of Working Conditions and Occupational Risk

The aggregate assessment of working conditions showed pronounced heterogeneity with respect to the structural divisions of the enterprise. Technological production units exhibited the least favorable occupational risk profile, while open-pit mining and auxiliary units had relatively lower percentages of high-risk workplaces.

Table 2 shows that high-risk workplaces accounted for 61.1% of jobs in technological units compared with 10.5% in open-pit mining units and 13.9% in auxiliary units. On the other hand, acceptable-risk workplaces were more common in open-pit mining units (47.4%) and auxiliary units (33.3%) than in technological units (22.2%). These results suggest that the occupational risk was not equally distributed in the enterprise but rather concentrated in technological production areas (Table 2).

### 3.2. Temporary Disability

Over the five-year observation period (2020–2024), a total of 4971 temporary disability cases and 61,472 lost working days were registered at the enterprise. The summary indicators for the full period were as follows: incidence rate (P1) = 42.85 cases per 100 employees; disability day rate (P2) = 529.9 days per 100 employees; mean episode duration (P3) = 12.37 days per case; and recurrence index (P4) = 27.16 cases per 100 employees.

Annual trends followed an undulating rather than a monotonic pattern. The highest temporary disability burden was recorded in 2022, with 1258 episodes and 16,028 lost working days, corresponding to 53.85 cases and 686.1 days per 100 employees. This peak, possibly influenced by the COVID-19 pandemic’s occupational health impact, was followed by a progressive decline and subsequent partial stabilization in the later years of the observation period.

### 3.3. Psychosocial Work Environment

The author-developed psychosocial work environment questionnaire demonstrated good internal consistency for the total scale, with Cronbach’s alpha = 0.82. The domain-level coefficients ranged from 0.76 to 0.84, indicating acceptable to good reliability across the three conceptual domains. Convergent validity was supported by a statistically significant positive correlation between the integral psychosocial adversity index and the total PSS-25 score (rho = 0.48; *p* < 0.001).

The EFA revealed a clear three-factor structure accounting for 68.4% of the common variance. The KMO value was 0.873, indicating meritorious sampling adequacy. Bartlett’s test rejected the null hypothesis of an identity matrix (χ^2^(210) = 3247.6, *p* < 0.001). After varimax rotation, the first factor (eigenvalue 6.81) explained 32.4% of the variance and loaded strongly on items related to workload norms, leadership satisfaction, and communication (loadings 0.52–0.75). The second factor (eigenvalue 4.37, 20.8% variance) captured organizational justice, pay fairness, and career development (loadings 0.47–0.72). The third factor (eigenvalue 3.18, 15.2% variance) comprised social benefits, medical support, and sickness assistance (loadings 0.63–0.80). All primary factor loadings exceeded 0.50, and cross-loadings were below 0.40, supporting construct validity.

The psychosocial indices clearly differentiated occupational groups across the enterprise. The most favorable profiles were observed among administrative personnel, whereas production unit employees—particularly workers in open-pit mining areas—reported significantly lower scores on the organizational justice and well-being subscales. Inter-group differences were statistically significant across all domains (*p* < 0.001). A comparative psychosocial profile of the studied occupational groups is presented in Table 3.

### 3.4. Occupational Stress

Occupational stress was assessed annually using the PSS-25 Industrial scale during the 2023–2025 occupational health examination waves. The annual subsamples included 133 employees in 2023, 133 in 2024, and 134 in 2025. The mean PSS-25 scores were 40.5 in 2023, 40.4 in 2024, and 41.9 in 2025, with a median value of 40 across all three years. These results indicate that the average stress level remained relatively stable over the observation period.

The categorical distribution provided additional information on the structure of the stress burden. Approximately half of the surveyed employees were classified as having moderate stress each year. The proportion of employees in the low-stress category declined from 21.8% in 2023 to 17.9% in 2025, while the proportion classified as high stress increased from 24.1% to 28.4%. The very high stress category remained relatively small, ranging from 3.7% to 4.5% annually (Table 4).

### 3.5. Integral Correlations

The main integrated finding was the convergence of hygienic, psychosocial, stress-related, and medical-statistical indicators. The Integral Index of Working Conditions (Iwc) showed positive correlations with temporary disability indicators P1–P4 (r = 0.35–0.50; *p* < 0.01). This suggested that less favorable working conditions were associated with a higher temporary disability burden at the enterprise or unit level.

Iwc was also positively correlated with total PSS-25 scores (r = 0.40; *p* < 0.01), indicating that poorer working conditions were associated with higher levels of occupational stress. Conversely, the Integral Health Index (Ihr) showed an inverse relationship with the integral psychosocial well-being index (r = −0.45; *p* < 0.01), such that more favorable psychosocial conditions corresponded to a more favorable health profile. A conceptual representation of these interrelationships is presented in Table 5.

A multidimensional linear regression model was fitted to test independent associations, adjusting for possible confounding. Model (dependent variable: PSS-25 stress score) included Iwc, integral psychosocial index, age, job tenure, and job category (production vs. administrative) as predictors. The model was significant (F(5, 386) = 47.8, *p* < 0.001) and explained 53% of the variance in occupational stress (adjusted R^2^ = 0.52). Iwc exhibited a significant positive independent effect (β = 0.31, 95% CI: 0.15–0.47, *p* = 0.001), as well as a significant negative independent effect for the integral psychosocial index (β = −0.39, 95% CI: −0.53 to −0.25, *p* < 0.001). Neither age (*p* = 0.22), job tenure (*p* = 0.59), nor job category (*p* = 0.29) were statistically significant, indicating that their effects on stress are in large part mediated by the working environment and psychosocial climate.

## 4. Discussion

We explored if an integrated assessment of work conditions, psychosocial work environment, occupational stress and temporary disability indicators in an industrial enterprise can offer a more informative occupational health risk profile. The findings further support the rationale that occupational risk evaluation within these settings should not be confined to hygienic and physical exposures alone. The profiles of the least desirable working conditions were all technological production units, and these units exhibited a relatively poor psychosocial profile and greater stress. This can be consistent with the occupational health literature that indicates psychosocial risk is not a substitute for traditional hazards but adds to the general burden of the individual worker [2,6,7,8,9,10,11,12].

An important contribution of this study is that the psychosocial work environment was not analyzed as a distinct perception-based construct. It was instead considered in combination with a formal assessment of workplace risk, occupational stress indexed on PSS-25 Industrial, and medical-statistical markers of temporary disability. Such an integrated approach enhances the practical relevance and impact of the findings, providing a better understanding of how psychosocial-related elements correspond to measurable work-disability indicators.

The psychometric results further validated the applicability of the author-developed psychosocial questionnaire in this industrial setting. Its internal consistency was acceptable to good, and the exploratory factor analysis supported the proposed three-domain structure of the instrument. The finding of a substantial correlation of the integral psychosocial adversity index with PSS-25 scores also supported convergent validity. At the same time, these results deserve to be read as preliminary validation evidence. The questionnaire should be considered a context-specific instrument. For the instrument to be adequately validated for wider use, confirmatory factor analysis (CFA) and external validation would be required in an independent industrial sample.

The associations observed between the Integral Index of Working Conditions (Iwc) and temporary disability indicators must be interpreted with caution. Because temporary disability indicators were available only at the aggregated enterprise or unit level, while psychosocial and stress data were obtained as individual constructs, direct causal inferences at the person-level are not possible. To avoid the risk of an ecological fallacy, we restricted the analysis of morbidity data to descriptive comparisons rather than predictive modeling. These unit-level morbidity trends serve to illustrate the broader occupational health context, indicating that adverse working conditions and health burdens usually co-occur, but they do not prove that one directly causes the other for individual workers.

Moreover, the non-synchronous nature of our datasets presents a major temporality limitation. The temporary disability data spanned a retrospective multi-year period (2020–2024), while occupational stress was measured cross-sectionally during annual medical examinations in 2023–2025. Because these timeframes do not perfectly align, the observed associations cannot and should not be interpreted causally or directionally. The morbidity data are explicitly framed as a retrospective occupational health backdrop of the enterprise, rather than a direct, measurable outcome of the currently recorded psychosocial conditions. The 2022 temporary disability peak needs careful interpretation as well. It may have been influenced by post-pandemic circumstances or accumulated illness; since these factors were not modeled, the 2022 peak remains a descriptive finding rather than a signal towards a particular causal mechanism.

The results of this work help explain practices within occupational health. Prevention should continue in high-risk technology units with engineering, sanitary-hygienic, and organizational controls. Nevertheless, the results imply that such measures should be augmented by a psychosocial intervention targeting managerial support, communication, perceived fairness, workload allocation, and the resources available for recovery.

The above limitations must be acknowledged. First, the psychosocial and stress components were based on voluntary participation, which may introduce self-selection bias. Second, the reliance on self-reported measures for both psychosocial factors and stress raises the potential for common method bias. However, procedural remedies (ensuring anonymity) and statistical checks (Harman’s single-factor test, where the first factor explained 26.3% of the variance) indicate that this bias is unlikely to significantly distort the findings. Nonetheless, future studies should incorporate more objective indicators to further mitigate this risk. Third, as noted, CFA and external validation of the questionnaire were not conducted. Fourth, although our multivariable regression model at the individual level successfully adjusted for key confounders (age, tenure, and gender), residual confounding due to unmeasured factors (e.g., education, marital status, baseline health behaviors) cannot be completely excluded. The non-significant influence of demographic covariates in the last model should not be taken as proof of no effect, but rather to show how they can exert effect indirectly in the work environment.

Lastly, longitudinal study designs with harmonized, person-level time windows in future studies are needed to determine the true mechanisms and strength of these associations. Overall, the findings provide evidence for a holistic assessment model for occupational health, which comprises working conditions, psychosocial environment, occupational stress, and health-related outcomes. This type of approach may potentially help prioritize preventive activities and better workplace management in industrial enterprises.

## 5. Conclusions

On a preventive basis, the current results provide evidence for an integrated approach to intervention. In high-risk units, engineering and sanitary–hygienic measures should be followed by targeted actions such as management support, fair labor relations, organizational communication, work scheduling, and recovery opportunities. This model is consistent with the constructs of health-promoting workplaces and psychosocial risk management with contemporary models [1,3].

The integrated assessment demonstrated substantial heterogeneity of occupational risk across the enterprise, with the most unfavorable profiles concentrated in technological production units. This supports the need for differentiated preventive strategies across occupational groups, rather than a uniform enterprise-wide approach.

The temporary disability burden was considerable during the five-year observation period, with 4971 eligible cases and 61,472 lost working days. These findings confirm the practical value of indicators P1–P4 as enterprise-level markers for monitoring workforce health and work-disability burden.

The author-developed psychosocial questionnaire demonstrated acceptable internal consistency, an interpretable three-factor structure, and convergent validity with PSS-25 Industrial scores. This supports its use as a context-specific instrument for psychosocial risk screening in industrial settings, although further confirmatory validation is required.

Occupational stress remained relatively stable at the population level from 2023 to 2025, but a persistent subgroup of workers with high or very high stress was identified. This group should be considered a priority target for psychosocial prevention and occupational health follow-up.

The observed associations between Iwc, temporary disability, occupational stress, and psychosocial well-being indicate that hygienic, psychosocial, stress-related, and medical-statistical indicators should be interpreted within a single occupational risk assessment framework. This approach may provide a practical basis for evidence-informed workplace health management in mining and processing enterprises.

## Figures and Tables

**Table 1:** Study population.

Characteristic	Production Units	Open-Pit Mining	Services	Administration	Total
n (%)	96 (24.5%)	193 (49.2%)	55 (14.0%)	48 (12.2%)	392 (100%)
Response Rate, %	72.8%	74.1%	71.3%	69.6%	73.0%
Age, M ± SD	42.3 ± 8.5	41.7 ± 9.2	39.8 ± 7.6	38.4 ± 6.9	40.8 ± 8.4
Tenure (years), M ± SD	12.4 ± 9.1	13.8 ± 10.3	11.5 ± 8.7	9.2 ± 6.8	12.1 ± 9.2

**Table 2:** Distribution of integrated occupational risk categories across enterprise unit groups.

Unit Group	Acceptable Risk	Elevated Risk	High Risk
Open-pit mining units	47.4%	42.1%	10.5%
Technological units	22.2%	16.7%	61.1%
Auxiliary units	33.3%	52.8%	13.9%

Note: Values are proportions of workplaces classified into integrated occupational risk categories.

**Table 3:** Psychosocial work environment scores by worker group.

Psychosocial Index	Technological Units (n = 96)	Open-Pit Units (n = 193)	Services (n = 55)	Administration (n = 48)	*p* Value
Social responsibility and support	70.0 ± 10.5 (95% CI 67.0–73.0)	65.0 ± 12.0 (95% CI 62.0–68.0)	75.0 ± 8.0 (95% CI 72.0–78.0)	80.0 ± 7.0 (95% CI 77.0–83.0)	<0.001
Social justice and organization of work	72.0 ± 12.0 (95% CI 68.0–76.0)	60.0 ± 15.0 (95% CI 55.0–65.0)	78.0 ± 9.0 (95% CI 75.0–81.0)	85.0 ± 8.0 (95% CI 81.0–89.0)	<0.001
Worker well-being	68.0 ± 11.0 (95% CI 65.0–71.0)	55.0 ± 14.0 (95% CI 51.0–59.0)	77.0 ± 10.0 (95% CI 74.0–80.0)	82.0 ± 9.0 (95% CI 79.0–85.0)	<0.001

**Table 4:** Distribution of occupational stress categories by year.

Year	Low Stress	Moderate Stress	High Stress	Very High Stress
2023	29 (21.8%)	66 (49.6%)	32 (24.1%)	6 (4.5%)
2024	27 (20.3%)	66 (49.6%)	35 (26.3%)	5 (3.8%)
2025	24 (17.9%)	67 (50.0%)	38 (28.4%)	5 (3.7%)

**Table 5:** Integrated working conditions and health profile by unit and tenure group.

Unit	Tenure	Iwc	Occupational Risk Category	Ihr	Health Status Category
Open-pit mining unit	≤5 years	1.8	Harmful working conditions, degree 2 (Class 3.2)	0.40	Moderate
Open-pit mining unit	5–10 years	2.2	Harmful working conditions, degree 3 (Class 3.3)	0.28	Favorable
Open-pit mining unit	>10 years	1.0	Harmful working conditions, degree 1 (Class 3.1)	0.22	Favorable
Gold-processing plant	≤5 years	1.5	Harmful working conditions, degree 2 (Class 3.2)	0.76	Unfavorable
Gold-processing plant	5–10 years	1.3	Harmful working conditions, degree 1–2 (Class 3.1–3.2)	0.37	Moderate
Gold-processing plant	>10 years	1.0	Harmful working conditions, degree 1 (Class 3.1)	0.65	Moderate
Auxiliary units	≤5 years	1.2	Harmful working conditions, degree 1 (Class 3.1)	0.29	Favorable
Auxiliary units	5–10 years	1.0	Harmful working conditions, degree 1 (Class 3.1)	0.75	Unfavorable
Auxiliary units	>10 years	0.0	Acceptable working conditions (Class 2)	0.91	Unfavorable

## Data Availability

The data presented in this study are available on request from the corresponding author. The data are not publicly available due to privacy and ethical restrictions related to the occupational health records of enterprise employees.

## Abbreviations

The following abbreviations are used in this manuscript:

PSS-25	Perceived Stress Scale (25-item version)
Iwc	Integral Index of Working Conditions
Ihr	Integral Health Index
ANOVA	Analysis of variance
EFA	Exploratory factor analysis
